# Cross-Talk between Gut Microbiota and the Heart: A New Target for the Herbal Medicine Treatment of Heart Failure?

**DOI:** 10.1155/2020/9097821

**Published:** 2020-04-06

**Authors:** Lin Li, Senjie Zhong, Bin Cheng, Hong Qiu, Zhixi Hu

**Affiliations:** ^1^The Domestic First-Class Discipline Construction Project of Chinese Medicine, Hunan University of Chinese Medicine, Changsha, Hunan, China; ^2^Institute of Traditional Chinese Medicine Diagnostics, Hunan University of Chinese Medicine, Changsha, Hunan, China; ^3^Post-Graduate School, Hunan University of Chinese Medicine, Changsha, Hunan, China

## Abstract

Heart failure (HF) is the severe and terminal stage of various heart diseases. A growing number of studies have suggested the potential clinical significance of gut microbiota in the pathophysiology of HF. Herbal medicine (HM) plays a role in rebalancing the composition of gut microbiota and is widely used in the prevention and treatment of HF. There are many similarities between intestinal microecology and the traditional Chinese medicine (TCM) theory, such as the holistic concept and the theory of the “heart's connection with the small intestine.” These similarities provide a theoretical basis for HM to prevent and treat diseases by regulating the intestinal flora and its metabolites. In this work, the cross-talk between gut microbiota and the heart is reviewed, and the relationship between TCM and gut microbiota is discussed. Based on the current literature and research, we hypothesize that the cross-talk between gut microbiota and the heart may offer a new therapeutic target for HF intervention.

## 1. Introduction

Heart failure (HF) is the terminal stage of all cardiac diseases, with high morbidity and mortality [1–3]. There has recently been increasing interest in studying the gut microbiota-heart interaction because the gut microbiota has been recognized as a barometer of human health [4]. Studies have shown that gut microbiota and its metabolites can directly participate in the normal physiological and metabolic activities of the human body and they can play a role in the occurrence and development of cardiovascular diseases through inflammation, immunity, and metabolism [5, 6]. The potential role of the gut in the pathophysiology of HF has recently attracted more and more attention. It has been shown that lowering the gut metabolism or changing the composition of gut microbiota may reduce the risk of HF. A growing number of studies support the role of the gut in the pathogenesis of HF in what is called “the gut hypothesis” [7].

Traditional Chinese medicine (TCM) has accumulated rich experience in the treatment of HF [8–14] and is commonly used as a complement to evidence‐based therapies for chronic and acute HF [15–17]. Chinese herbal medicine combined with conventional medicine treatment could improve chronic heart failure (CHF) patients' quality of life (QoL) [18]. The TCM use may be driven by a wide‐scale availability, even in Western medicine hospitals; studies show that three fourths of the patients with HF receive TCM treatment during their hospitalization for HF, and almost all hospitals use TCM treatment [19]. To explore the underlying action mechanisms of Chinese herbs, extensive research has been conducted. For instance, Yangxinkang tablets can effectively improve the cardiac function, symptoms, physical signs, and life quality for CHF patients in stage C [20]. Qiliqiangxin capsules can effectively enhance the cardiac function, symptoms, and physical signs of CHF patients without obvious toxicity or side effects [21]. Tongbu Xinbao capsules can relieve the symptoms and physical signs of chronic congestive HF patients without side effects or toxicity [22]. Shencaotongmai granules can improve the left ventricular ejection fraction and the symptoms of chronic cardiac failure patients, showing a good and safe curative effect [23]. A research work was conducted on the systematic evaluation of the abovementioned TCM regarding the safety and curative effect on HF, impacts of the medicine ingredients on the cardiovascular system, and their potential mechanisms. The results suggested that these TCM medications might be effective in improving the cardiac remodeling and function in patients with HF, with a good safety profile [17]. In addition, other studies have reviewed the published clinical evaluation and experimental studies about using TCM medications to treat heart failure, proving that TCM medications show the effects of antifibrosis, anti-inflammation, antioxidation, antiapoptosis, proangiogenesis, and metabolism regulatory. TCM is thus expected to become an effective way to treat HF [16].

**Figure 1:**
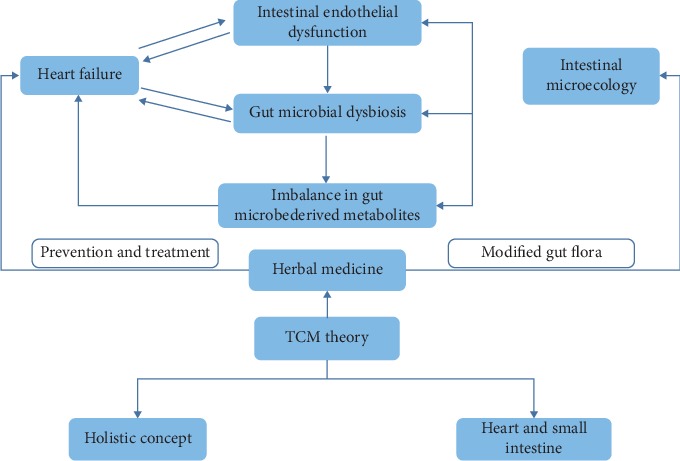
Cross-talk between gut microbiota and the heart, a new target for HM treatment of HF. In patients with HF, microcirculatory disturbances result in intestinal epithelial dysfunction. On the other hand, HF is associated with gut microbiota dysbiosis and the imbalance of gut microbe-derived metabolites. Evidence has revealed the role of HM in modulating the gut microbiota, and HM is widely used in the prevention and treatment of HF. There are many similarities between intestinal microecology and the TCM theory, such as the holistic concept and the theory of the heart's connection with the small intestine.” These similarities provide a theoretical basis for HM to prevent and treat diseases by regulating the intestinal flora and its metabolites.

Studies have shown that some HMs exert their effects on the diseases by modulating gut microbiota and its metabolites [24, 25] and are widely used in the prevention and treatment of HF. There are many similarities between intestinal microecology and the TCM theory, such as the holistic concept and the theory of the “heart's connection with the small intestine.” These similarities provide a theoretical basis for HM to prevent and treat diseases by regulating intestinal microecology. This suggests that the cross-talk between gut microbiota and the heart may become a new therapeutic target for HF intervention [26]; the connection is shown in Figure 1. Novel therapeutic strategies are targeting the gut microbial metabolic pathways and/or metabolites based on TCM, which have the potential to modulate the cardiovascular disease (CVD) susceptibility and prevent progression to HF.

In this paper, we present a review of the cross-talk between gut microbiota and the heart in HF and discuss the relationship between TCM and gut microbiota from the perspective of herbal medicine and the TCM theory, including the holistic concept and the connection of the heart and small intestine.

## 2. Gut Microbiota and TCM

HM plays a role in rebalancing the composition of gut microbiota and is widely used as a complement to the evidence‐based therapies for HF. The similarities between intestinal microecology and the TCM theory provide a theoretical basis for HM to prevent and treat diseases by regulating the intestinal flora and its metabolite, such as the holistic concept and the theory of the “heart's connection with the small intestine.”

### 2.1. Gut Microbiota and the TCM Theory

#### 2.1.1. Holistic Concept of TCM

TCM research focuses on the normal physiological activities and disease states as a whole perspective of the human body. There are two parts in the holistic concept. First, the holistic concept of TCM emphasizes the unity of the human body itself and its indivisibility from the natural environment. Second, the gut interacts with other organs [27].

Over the long-term process of evolution, intestinal microecology has also formed a system of interdependence and mutual restriction between the flora, the host, and the environment through individual adaptation and natural selection. Intestinal microecology and the human body symbiotically coexist and interconnect with each other in terms of the structure, physiology, and pathology, revealing that the human body is an organic whole. Besides being controlled by genetic factors, the changes in intestinal microorganisms are also regulated by natural and social environmental factors. Additionally, the changes in lifestyle, especially dietary structure, are also typical factors affecting intestinal microecology, and lifestyle itself is closely related to the environment. The correlation between intestinal microecology and diet, environment, and other factors reflects the correspondence and close relationship between humans, nature, and social environment in traditional Chinese medicine.

In addition, the gut interacts with other organs. Well-characterized bidirectional communication channels exist between the gut and the brain, known as the brain-gut axis. These channels regulate neural, endocrinal, and inflammatory mechanisms through the permeability of the intestinal wall and the blood-brain barrier [28]. Similarly, studies have shown that intestinal microecology can also affect the occurrence of diseases through the gut-kidney axis and gut-liver axis [29, 30].

The study of intestinal microecology revealed the integrity of the human body in TCM from multiple aspects.

#### 2.1.2. The “Heart and Small intestine” Theory

The theory of “the heart's connection with the small intestine” comes from the text of “Miraculous Pivot.” It describes a close physiological and pathological relationship between them through the meridian [31].

From a physiological perspective, the heart is thought to dominate blood and vessels by the warming function of heart-yang and nourishing function of heart-blood, which contribute to the “digestive function” of the small intestine. On the other hand, the small intestine could separate the clear from turbid in the food. The clear refers to the food essence, which is transported and distributed to the heart by the spleen and transformed into blood in order to nourish the heart, while the turbid is transported into the large intestine and urinary bladder. In the theory of channels and collaterals, the heart meridian of Hand-Shao yin pertains to the heart and connects with the small intestine, while the small intestine meridian pertains to the small intestine and connects with the heart. Thus, heart diseases could be transmitted to the small intestine by the meridian.

The text of the Golden Mirror of Medicine says “The heart and small intestine is in an interior-exterior dyad. So this may manifest the symptom as oliguria with yellow or red urine and dysuria, odynuria, pyretic stranguria, a red tongue and sores in the mouth in the condition that the excessive fire of the heart transmit to the small intestine.” “The disease of small intestine may also transmit to heart. For example the dysfunction of small intestine being concerned with the thick and turbid body fluid can lead excessive-fluid to influence the heart.” The function of “heart housing the mind” would be influenced by the condition that the heat of the small intestine hits the heart. The eighth volume of Wang's medical preservation says “The upgoing of the heat of small intestine makes agrypnia.”

The theory of “the heart's connection with the small intestine” of TCM also coincides with the gut hypothesis. Based on this theory and the gut microbiota research progress of modern medicine, we think that gut microbiota is the biological foundation for a normal development of the intestinal physiological function. The findings show that gut microbiota develops an important role in digestion, absorption, and excretion of human nutrient substances. On the one hand, it promotes the absorption of nutrient substances in the blood. On the other hand, it assists to excrete the metabolites generated in the digestion process in vitro [32]. This corresponds to the physiological functions of “containing and digesting” and “separating the clear from the turbid” in the theory of TCM.

In summary, the theory of “the heart's connection with the small intestine” is the basis for the hypothesis of “treating the heart-disease by regulating the enteric microorganisms” [33–35].

### 2.2. Gut Microbiota and Herbal Medicine

Based on the revealed role of HM in modulating gut microbiota [36–39], using HM to target the “microbiota-metabolism-immunity” axis could be a possible therapy for CVD [40]. HM has a bidirectional regulatory effect on gut microbiota, and it can promote the proliferation of beneficial bacteria and inhibit the growth of harmful ones.

It was shown that the HM products can interact with gut microbiota when they enter the gastrointestinal tract, and this interaction was summarized in three aspects. First, HM can modulate the composition of gut microbiota. Second, HM can modulate the metabolism of gut microbiota. Finally, gut microbiota can transform the HM compounds [36, 41–43]. These interactions can generate a series of metabolites with potential extensive effects on the hosts.

Studies have revealed the link between gut microbiota and heart failure, mainly through intestinal barrier damage and bacterial translocation to induce inflammation and immune response [44]. HM can regulate intestinal microflora, protect the intestinal mucosal barrier, restore the intestinal microbial diversity, and enhance the immune function [45–48].

Current studies show that HM mainly regulates intestinal flora by monomers or formulae. First, some HMs that contain polysaccharides have a probiotic-like effect and can stimulate the growth of symbiotic beneficial bacteria, such as *Lactobacillus*, *Bifidobacterium*, and *Bacteroides* [42]. These beneficial bacteria could prevent pathogenic bacteria from invading. For example, astragaloside can regulate the intestinal microenvironment disorders, increasing the abundance of *Bifidobacterium*, *Brucella*, and *Clostridium* [49], while Ginseng polysaccharide can improve the absorption of ginsenosides and promote the growth of *Lactobacillus* and *Bacteroides* [50]. In addition, HM can regulate the intestinal mucosal barrier to prevent bacterial translocation. Research has shown that Xiao-Qing-Long-Tang could prevent cardiomyocyte hypertrophy and fibrosis, and it could improve the intestinal mucosal histology by regulating the composition of gut microbiota [51]. Tong-Xie-Yao-Fang can effectively improve the intestinal permeability and enhance the intestinal mucosal barrier function [52]. Cordyceps polysaccharide can improve the intestinal flora and integrity and reduce the metabolic endotoxins and inflammation [53]. Moreover, HM can influence intestinal immunity through the regulation of gut microbiota. The intestinal tract is the most abundant immune organ of the human body, which undertakes important defense tasks [54]. HM can regulate the body's immunity to prevent and treat the intestinal mucosal damage. For instance, *Dendrobium huoshanense* polysaccharide could regulate the intestinal immunological barrier function by stimulating the production of cytokines and functional development of the cells of the immune system [55]. *Astragalus membranaceus* can reduce the intestinal mucosal damage and promote tissue repair by inhibiting the expression of inflammatory cytokine [56]. Therefore, using HM to regulate gut microbiota could be a possible therapy for HF.

## 3. Gut Microbiota and CVD

CVD continues to be the leading cause of death and disability in modern societies [57], accounting for over one-third of all deaths worldwide with an annual cost of nearly $1 trillion [57, 58]. In light of these statistics, it is of high biomedical importance to elucidate the underlying causes of CVD and identify potential therapeutic targets for its prevention and treatment. Recent evidence has indicated that gut microbiota is linked to the development and progression of CVD [5, 59–62].

The human intestinal tract is symbiotic with a large number of a wide variety of microorganisms, collectively known as the gut microbiota. It has been estimated that microbes in our bodies collectively make up to 100 trillion cells, tenfold the number of human cells, and it is suggested that they encode a hundredfold more unique genes than our own genome [63]. Most microorganisms live in the gut, which has a profound impact on the human physiology and nutrition and is crucial for human life [64, 65], since this microbiota plays an important role in the human energy metabolism, material absorption, immune regulation, and other aspects [66]. When the gut dysbiosis takes place, causing inflammation and metabolic disorders, this promotes the development of CVD. Gut microbiota-host interactions occur through many pathways, including the trimethylamine-N-oxide (TMAO) [67–69], short-chain fatty acids (SCFA) [70, 71], and primary and secondary bile acids (BAs) [72, 73].

The last decade has seen significant advances in our understanding of the role of the microbiome in regulating CVD, including hypertension, atherosclerosis, and HF. Therefore, gut microbiota can be a new target for the treatment of CVD [74]. Further clinical and animal experiments are being conducted to determine the intestinal bacterial structure types and major molecular pathway mechanisms of cardiovascular diseases, which can be used for the intervention and treatment at the early stage of the disease, thus slowing down or preventing its development.

## 4. Cross-Talk between Gut Microbiota and the Heart

HF has long been recognized to be associated with altered intestinal functionality [75, 76]. The gastrointestinal system has been implicated in the pathogenesis of HF according to a growing number of studies that support the role of the gut in HF pathogenesis under “the gut hypothesis” [7]. Thus, the novel concept of a heart-gut axis may lead to new insights and breakthroughs in the development of innovative diagnostic and therapeutic approaches for HF [4, 26, 77].

### 4.1. Intestinal Endothelial Dysfunction

The intestinal barrier function is usually maintained by well-balanced intestinal microbial communities, intact mucosal tight junctions, normal mucosal immunity, and normal sodium homeostasis. When visceral circulatory congestion happens during HF, bacterial translocation can occur due to the altered intestinal barrier function, intestinal pathogens then increase and the host defense function gets damaged, the intestinal wall blood flow decreases, and morphological changes occur with an increase in permeability, leading to endotoxemia and then to systemic inflammation [7, 78]. Cardiac cachexia is associated with intestinal congestion. Regardless of the HF stage and cardiac function, chronic heart failure patients have thicker intestinal walls than in noncachexia patients [77]. The intestinal epithelial cells may be impaired by intestinal ischemia, and epithelial dysfunction further impairs the absorption of sugar, protein, and fat, which may have an adverse effect on the development of cachexia and further complicated cases of advanced HF.

It has been reported that patients with congestive heart failure (CHF) may have intestinal overgrowth of pathogenic bacteria and increased *Candida* genera and intestinal permeability, which are associated with clinical disease severity, venous blood congestion, inflammation, increased intestinal permeability in patients with HF, and an increased number of bacteria and fungi in feces [79]. This suggests that maintaining the normal functionality of the intestinal barrier may be a new target in the treatment of HF.

Additionally, circulatory adaptation in CHF patients due to myocardial dysfunction may cause microcirculatory injury, leading to the destruction of the intestinal barrier and exacerbating inflammation [80, 81]. These observations suggest that a better understanding of the regulation of the intestinal barrier function may develop the intestinal wall in HF treatment.

### 4.2. Gut Microbial Dysbiosis

Gut microbiota dysbiosis exists in CHF. Lowering the intestinal metabolism or changing the composition of intestinal flora may reduce the risk of HF, while the imbalance of gut flora promotes the occurrence and development of HF [80]. A recent study showed that the composition of gut microbiota in CHF was significantly different from that of healthy controls. Using metagenomics and metabolomics, fecal and plasma samples from 53 CHF patients and 41 healthy controls were analyzed. The results showed a decrease in *Faecalibacterium prausnitzii* and an increase in *Ruminococcus gnavus* in CHF patients; an imbalance of the gut microbes was also observed [82]. 16S ribosomal RNA gene sequencing of fecal samples obtained from 12 HF patients and 12 age-matched healthy control (HC) subjects has also shown that HF is associated with dysbiosis in gut microbiota. On the other hand, older HF patients had diminished proportions of Bacteroidetes and larger quantities of Proteobacteria compared with younger HF patients [83]. These results suggest that patients with HF have a significantly altered intestinal microbiota. Another supporting study showed that hypertension and HF were prevented in hypertensive mice by changing the gut microbiota through high-fiber diet and acetate supplementation [84].

### 4.3. Imbalance of Gut Microbe-Derived Metabolites

The imbalance of gut microbe-derived metabolites has also been shown to contribute to HF, such as the trimethylamine-N-oxide (TMAO), BAs, and short-chain fatty acids (SCFAs).

Trimethylamine-N-oxide (TMAO) is derived from the metabolites of the gut microbiota from specific dietary nutrients. Animal liver, red meat, egg yolk, deep-sea fish, wheat bran, and other common foods are rich in choline, betaine, and L-carnitine, and these substances contain trimethylamine (TMA) structures which will generate TMA after the intestinal flora metabolism [85]. Then, TMA will enter into the liver through blood circulation and will be oxidized and metabolized into TMAO by flavin monooxygenase (FMO) [69, 86, 87]. TMAO is linked to a higher risk of death and HF-related death, and a combination of TMAO and NT-proBNP could provide additional prognostic information [88]. Systematic review and meta-analysis also demonstrated a positive dose-dependent relationship between TMAO plasma levels and increased cardiovascular risk and mortality [89]. A study published in 2016 examined the relationship between fasting plasma TMAO and all-cause mortality over a 5-year follow-up in 720 patients with stable HF. The results revealed that TMAO levels in HF patients were significantly higher than the cases without HF, and elevated TMAO levels portended higher long-term mortality risk independent of the traditional risk factors and cardiorenal indexes [7]. Further animal studies confirmed a causal relationship between TMAO and HF susceptibility, which is not just a correlation [90].

BAs are currently recognized as signaling molecules, and studies have indicated that they affect the cardiovascular function [91]. A cross-sectional research revealed that the ratio of secondary to primary BAs was increased in patients with chronic heart failure, and this ratio was considered to be associated with a reduced overall survival in a univariate analysis [92]. The discovery of bile acid-responsive receptors strongly enhances the cognition of the relationship between BAs and HF, especially concerning the Farnesoid X Receptor (FXR) and G-protein Coupled Bile Acid Receptor 1 (TGR5). A study showed that FXR and TGR5, which are expected to become the latest target of HF treatment, are closely related to inflammation, myocardial function, and hemodynamic stress [93, 94].

Short-chain fatty acids (SCFAs), including acetic acid, propionic acid, and butyric acid, mainly belong to the fatty acids with a carbon number of 2 to 6. A few kinds of SCFAs receptors have been recently reported, such as Olfr78, the olfactory receptor of protein G-linked receptor (GPR) family [95]. It is believed that Olfr78 is related to hormone secretion and blood pressure regulation [96]. Related studies show that SCFAs participate in the energy metabolism of the host, and the high SCFAs content in the feces indicates a high risk of hypertension and heart metabolic diseases [97]. Other studies show that SCFAs are closely related to atherosclerosis [98]. Supplementing butyric acid in the diet can inhibit the atherosclerotic lesions of ApoE knock-out mice by reducing the macrophage migration rate, increasing the collagen deposition and plaque stability [99]. The current research results show that the SCFAs disorder may lead to the occurrence of hypertension, atherosclerosis, and other cardiovascular diseases. Therefore, the regulation of the SCFAs disorder is expected to become a new treatment target of these diseases.

## 5. Future Perspectives

Emerging evidence supports a novel link between the gut microbiota and HF. On the contrary, there are many similarities between intestinal microecology and TCM theories, and the gut hypothesis also coincides with the TCM theory of “the heart's connection with the small intestine.” This has led to the hypothesis that the cross-talk between gut microbiota and the heart may become a new target for HM treatment in HF. More animal and clinical trials are needed to systematically understand how the gut microbes can convert diet or TCM into metabolites that interact with surrounding tissues and organs.

The development of a new generation of nucleic acid sequencing technology and metagenomic technology has greatly promoted the research of intestinal microbiome in the CVD field. Genome sequencing is not only able to obtain the composition and functional gene information of bacterial flora, but it can also identify specific bacterial flora that is related to certain diseases. The new generation of ribonucleic acid sequencing technology combined with metagenomic technology is conducive to the discovery of the changes in intestinal flora in TCM syndrome differentiation and searching for potential metabolic markers. TCM can treat diseases by regulating the gut microbiota to change the metabolism of the body. Therefore, the identification of the gut microbiota and their metabolites can be significant in developing individualized intervention strategies.

Gut microbiota may represent a new target of HM regulation in HF based on the cross-talk between gut microbiota and heart; the intervention treatment of the host metabolic diseases may provide new insights into the prevention and treatment of HF.

## References

[1] Metra M., Teerlink J. R. (2017). Heart failure. *The Lancet*.

[2] Bui A. L., Horwich T. B., Fonarow G. C. (2011). Epidemiology and risk profile of heart failure. *Nature Reviews Cardiology*.

[3] Yancy C. W., Jessup M., Bozkurt B. (2017). 2017 ACC/AHA/HFSA focused update of the 2013 ACCF/AHA guideline for the management of heart failure: a report of the American College of Cardiology/American Heart Association Task Force on clinical practice guidelines and the Heart Failure Society of America. *Journal of Cardiac Failure*.

[4] Bu J., Wang Z. (2018). Cross-talk between gut microbiota and heart via the routes of metabolite and immunity. *Gastroenterology Research and Practice*.

[5] Tang W. H. W., Kitai T., Hazen S. L. (2017). Gut microbiota in cardiovascular health and disease. *Circulation Research*.

[6] Brandsma E., Kloosterhuis N. J., Koster M. (2019). A proinflammatory gut microbiota increases systemic inflammation and accelerates atherosclerosis. *Circulation Research*.

[7] Tang W. H. W., Wang Z., Fan Y. (2014). Prognostic value of elevated levels of intestinal microbe-generated metabolite trimethylamine-N-oxide in patients with heart failure. *Journal of the American College of Cardiology*.

[8] Du Z., Wen R., Liu Q. (2019). 1H NMR-based dynamic metabolomics delineates the therapeutic effects of Baoyuan decoction on isoproterenol-induced cardiac hypertrophy. *Journal of Pharmaceutical and Biomedical Analysis*.

[9] Huang L., Cai H., Zhuang J. (2018). Fuling Sini decoction for patients with chronic heart failure: a protocol for a systematic review and meta-analysis. *Medicine (Baltimore)*.

[10] Ren Y., Chen X., Li P. (2019). Si-Miao-Yong-An decoction ameliorates cardiac function through restoring the equilibrium of SOD and NOX2 in heart failure mice. *Pharmacological Research*.

[11] Tang Q., Wang Y., Li K. (2018). Zhenwu decoction for chronic heart failure: protocol for a systematic review and meta-analysis. *Medicine*.

[12] Yan X., Wu H., Ren J. (2018). Shenfu Formula reduces cardiomyocyte apoptosis in heart failure rats by regulating microRNAs. *Journal of Ethnopharmacology*.

[13] Zhao Y., Jiang Y., Chen Y. (2019). Dissection of mechanisms of Chinese medicinal formula Si-Miao-Yong-An decoction protects against cardiac hypertrophy and fibrosis in isoprenaline-induced heart failure. *Journal of Ethnopharmacology*.

[14] Xian S., Yang Z., Lee J. (2016). A randomized, double-blind, multicenter, placebo-controlled clinical study on the efficacy and safety of Shenmai injection in patients with chronic heart failure. *Journal of Ethnopharmacology*.

[15] Tsai M.-Y., Hu W.-L., Lin C.-C. (2017). Prescription pattern of Chinese herbal products for heart failure in Taiwan: a population-based study. *International Journal of Cardiology*.

[16] Wang Y., Wang Q., Li C. (2017). A review of Chinese herbal medicine for the treatment of chronic heart failure. *Current Pharmaceutical Design*.

[17] Hao P., Jiang F., Cheng J., Ma L., Zhang Y., Zhao Y. (2017). Traditional Chinese medicine for cardiovascular disease: evidence and potential mechanisms. *Journal of the American College of Cardiology*.

[18] Li Y.-L., Ju J.-Q., Yang C.-H., Jiang H.-Q., Xu J.-W., Zhang S.-J. (2014). Oral Chinese herbal medicine for improvement of quality of life in patients with chronic heart failure: a systematic review and meta-analysis. *Quality of Life Research*.

[19] Yu Y., Spatz E. S., Tan Q. (2019). Traditional Chinese medicine use in the treatment of acute heart failure in western medicine hospitals in China: analysis from the China PEACE retrospective heart failure study. *Journal of the American Heart Association*.

[20] Xian S.-x., Yang Z.-q., Ren P.-h. (2015). Effect of yangxinkang tablets on chronic heart failure: a multi-center randomized double-blind placebo-controlled trial. *Chinese Journal of Integrative Medicine*.

[21] Li X., Zhang J., Huang J. (2013). A multicenter, randomized, double-blind, parallel-group, placebo-controlled study of the effects of Qili Qiangxin capsules in patients with chronic heart failure. *Journal of the American College of Cardiology*.

[22] Jia J. B., Fan R. H. (2018). Clinical efficacy of Tongbu Xinbao capsule on Qi deficiency blood stasis and water flux type chronic heart failure. *Chinese Journal of Experimental Traditional Medical Formulae*.

[23] Wang C., Zhang Y., Gong L. H. (2012). Treatment of chronic heart failure with Shencaotongmai granule: a multi-center, double-blinded, randomized, parallel-controlled trial. *Chinese Journal of Integrated Traditional and Western Medicine*.

[24] Chang C. J., Lin C. S., Lu C. C. (2015). Ganoderma lucidum reduces obesity in mice by modulating the composition of the gut microbiota. *Nature Communications*.

[25] Lyu M., Wang Y. F., Fan G. W., Wang X. Y., Xu S. Y., Zhu Y. (2017). Balancing herbal medicine and functional food for prevention and treatment of cardiometabolic diseases through modulating gut microbiota. *Frontiers in Microbiology*.

[26] Kamo T., Akazawa H., Suzuki J.-i., Komuro I. (2017). Novel concept of a heart-gut axis in the pathophysiology of heart failure. *Korean Circulation Journal*.

[27] Xu Z., Liu T., Zhou Q., Chen J., Yuan J., Yang Z. (2019). Roles of Chinese medicine and gut microbiota in chronic constipation. *Evidence-Based Complementary and Alternative Medicine*.

[28] Osadchiy V., Martin C. R., Mayer E. A. (2019). The gut-brain Axis and the microbiome: mechanisms and clinical implications. *Clinical Gastroenterology and Hepatology*.

[29] Yang T., Richards E. M., Pepine C. J., Raizada M. K. (2018). The gut microbiota and the brain-gut-kidney axis in hypertension and chronic kidney disease. *Nature Reviews Nephrology*.

[30] Milosevic I., Vujovic A., Barac A. (2019). Gut-liver axis, gut microbiota, and its modulation in the management of liver diseases: a review of the literature. *International Journal of Molecular Sciences*.

[31] Guo Z. Y., Liu Y., Gao Y. P., Peng L., Zhang Q. Y., Li J. (2017). Origin and development of the theory of “the heart and the small intestine sharing a paired relationship”. *Chinese Medicine*.

[32] Jonsson A. L., Bäckhed F. (2017). Role of gut microbiota in atherosclerosis. *Nature Reviews Cardiology*.

[33] Liu Y. W., Liu Z. Y. (2018). The relationship between intestinal flora and coronary heart disease based on the theory of “heart and small intestine”. *Lishizhen Medicine and Materia Medica Research*.

[34] LV X. M., Song N., Jia L. Q., Yang G. L. (2018). The relationship between intestinal flora and coronary heart disease was discussed based on the theory of “heart and the small intestine” in Neijing. *Lishizhen Medicine and Materia Medica Research*.

[35] Qu H., Jiang Z. H., Yang Q. N., Zhang X. Y., Tang Y. Z., Gao Z. H. (2018). Discussion on the association between atherosclerosis and intestinal microenvironment based on the theory of “heart connecting with small intestine”. *Chinese Medicine*.

[36] Feng W., Ao H., Peng C., Yan D. (2019). Gut microbiota, a new frontier to understand traditional Chinese medicines. *Pharmacological Research*.

[37] Yue S.-J., Wang W.-X., Yu J.-G. (2019). Gut microbiota modulation with traditional Chinese medicine: a system biology-driven approach. *Pharmacological Research*.

[38] Sang T. T., Guo C. J., Guo D. D., Wang X. Y. (2018). Effect of traditional Chinese medicine in inhibiting obesity and inflammatory diseases by regulating gut microbiota. *Zhongguo Zhong Yao Za Zhi*.

[39] Nie Q., Chen H., Hu J., Fan S., Nie S. (2019). Dietary compounds and traditional Chinese medicine ameliorate type 2 diabetes by modulating gut microbiota. *Critical Reviews in Food Science and Nutrition*.

[40] Anlu W., Xu H., Chen K. J. (2019). Using herbal medicine to target the “microbiota-metabolism-immunity” axis as possible therapy for cardiovascular disease. *Pharmacological Research*.

[41] Wang X.-M., Li X.-B., Peng Y. (2017). Impact of Qi -invigorating traditional Chinese medicines on intestinal flora: a basis for rational choice of prebiotics. *Chinese Journal of Natural Medicines*.

[42] Xu J., Chen H.-B., Li S.-L. (2017). Understanding the molecular mechanisms of the interplay between herbal medicines and gut microbiota. *Medicinal Research Reviews*.

[43] Yang Y., Chen G., Yang Q. (2017). Gut microbiota drives the attenuation of dextran sulphate sodium-induced colitis by Huangqin decoction. *Oncotarget*.

[44] Zabell A., Tang W. H. (2017). Targeting the microbiome in heart failure. *Current Treatment Options in Cardiovascular Medicine*.

[45] Lin Z., Ye W., Zu X. (2018). Integrative metabolic and microbial profiling on patients with Spleen-yang-deficiency syndrome. *Scientific Reports*.

[46] Shen H., Gao X.-J., Li T. (2018). Ginseng polysaccharides enhanced ginsenoside Rb1 and microbial metabolites exposure through enhancing intestinal absorption and affecting gut microbial metabolism. *Journal of Ethnopharmacology*.

[47] Li X. W., Chen H. P., He Y. Y. (2018). Effects of rich-polyphenols extract of Dendrobium loddigesii on anti-diabetic, anti-inflammatory, anti-oxidant, and gut microbiota modulation in db/db mice. *Molecules*.

[48] Jia Q., Wang L., Zhang X. (2020). Prevention and treatment of chronic heart failure through traditional Chinese medicine: role of the gut microbiota. *Pharmacological Research*.

[49] Xu N., Kan P., Yao X. (2018). Astragaloside IV reversed the autophagy and oxidative stress induced by the intestinal microbiota of AIS in mice. *Journal of Microbiology*.

[50] Zhou S. S., Xu J., Zhu H. (2016). Gut microbiota-involved mechanisms in enhancing systemic exposure of ginsenosides by coexisting polysaccharides in ginseng decoction. *Scientific Reports*.

[51] Zhou G. F., Jiang Y. H., Ma D. F. (2019). Xiao-qing-long Tang prevents cardiomyocyte hypertrophy, fibrosis, and the development of heart failure with preserved ejection faction in rats by modulating the composition of the gut microbiota. *Biomed Research International*.

[52] Hou Q., Huang Y., Zhu Z. (2019). Tong-Xie-Yao-Fang improves intestinal permeability in diarrhoea-predominant irritable bowel syndrome rats by inhibiting the NF-*κ*B and notch signalling pathways. *BMC Complementary and Alternative Medicine*.

[53] Wu T.-R., Lin C.-S., Chang C.-J. (2019). Gut commensal parabacteroides goldsteinii plays a predominant role in the anti-obesity effects of polysaccharides isolated from Hirsutella sinensis. *Gut*.

[54] Singh H., Yu Y., Suh M.-J. (2017). Type 1 diabetes: urinary proteomics and protein network analysis support perturbation of lysosomal function. *Theranostics*.

[55] Xie S.-Z., Liu B., Ye H.-Y. (2019). Dendrobium huoshanense polysaccharide regionally regulates intestinal mucosal barrier function and intestinal microbiota in mice. *Carbohydrate Polymers*.

[56] Cui Y., Wang Q., Sun R. (2018). Astragalus membranaceus (Fisch.) Bunge repairs intestinal mucosal injury induced by LPS in mice. *BMC Complementary and Alternative Medicine*.

[57] Jones D. S., Podolsky S. H., Greene J. A. (2012). The burden of disease and the changing task of medicine. *New England Journal of Medicine*.

[58] Fuster V. (2014). Global burden of cardiovascular disease: time to implement feasible strategies and to monitor results. *Journal of the American College of Cardiology*.

[59] Garcia-Rios A., Torres-Pena J. D., Perez-Jimenez F., Perez-Martinez P. (2017). Gut microbiota: a new marker of cardiovascular disease. *Current Pharmaceutical Design*.

[60] Peng J., Xiao X., Hu M., Zhang X. (2018). Interaction between gut microbiome and cardiovascular disease. *Life Sciences*.

[61] Singh V., Yeoh B. S., Vijay-Kumar M. (2016). Gut microbiome as a novel cardiovascular therapeutic target. *Current Opinion in Pharmacology*.

[62] Chong-Nguyen C., Duboc H., Sokol H. (2017). The gut microbiota, a new cardiovascular risk factor?. *La Presse Médicale*.

[63] Almeida A., Mitchell A. L., Boland M. (2019). A new genomic blueprint of the human gut microbiota. *Nature*.

[64] Qin J., Li R., Raes J. (2010). A human gut microbial gene catalogue established by metagenomic sequencing. *Nature*.

[65] Zhernakova A., Kurilshikov A., Bonder M. J. (2016). Population-based metagenomics analysis reveals markers for gut microbiome composition and diversity. *Science*.

[66] Sonnenburg J. L., Bäckhed F. (2016). Diet-microbiota interactions as moderators of human metabolism. *Nature*.

[67] Koeth R. A., Levison B. S., Culley M. K. (2014). *γ*-Butyrobetaine is a proatherogenic intermediate in gut microbial metabolism of L -carnitine to TMAO. *Cell Metabolism*.

[68] Zhu W., Gregory J. C., Org E. (2016). Gut microbial metabolite TMAO enhances platelet hyperreactivity and thrombosis risk. *Cell*.

[69] Wang Z., Klipfell E., Bennett B. J. (2011). Gut flora metabolism of phosphatidylcholine promotes cardiovascular disease. *Nature*.

[70] Ohira H., Tsutsui W., Fujioka Y. (2017). Are short chain fatty acids in gut microbiota defensive players for inflammation and atherosclerosis?. *Journal of Atherosclerosis and Thrombosis*.

[71] Hashemi Z., Fouhse J., Im H. S., Chan C. B., Willing B. P. (2017). Dietary pea fiber supplementation improves glycemia and induces changes in the composition of gut microbiota, serum short chain fatty acid profile and expression of mucins in glucose intolerant rats. *Nutrients*.

[72] Islam K. B. M. S., Fukiya S., Hagio M. (2011). Bile acid is a host factor that regulates the composition of the cecal microbiota in rats. *Gastroenterology*.

[73] Swales K. E., Moore R., Truss N. J. (2012). Pregnane X receptor regulates drug metabolism and transport in the vasculature and protects from oxidative stress. *Cardiovascular Research*.

[74] Vinje S., Stroes E., Nieuwdorp M., Hazen S. L. (2014). The gut microbiome as novel cardio-metabolic target: the time has come. *European Heart Journal*.

[75] Anker S. D., Egerer K. R., Volk H.-D., Kox W. J., Poole-Wilson P. A., Coats A. J. S. (1997). Elevated soluble CD14 receptors and altered cytokines in chronic heart failure. *The American Journal of Cardiology*.

[76] Krack A., Sharma R., Figulla H. R., Anker S. D. (2005). The importance of the gastrointestinal system in the pathogenesis of heart failure. *European Heart Journal*.

[77] Sandek A., Anker S., Haehling S. (2009). The gut and intestinal bacteria in chronic heart failure. *Current Drug Metabolism*.

[78] Sandek A., Bjarnason I., Volk H.-D. (2012). Studies on bacterial endotoxin and intestinal absorption function in patients with chronic heart failure. *International Journal of Cardiology*.

[79] Pasini E., Aquilani R., Testa C. (2016). Pathogenic gut flora in patients with chronic heart failure. *JACC: Heart Failure*.

[80] Sandek A., Bauditz J., Swidsinski A. (2007). Altered intestinal function in patients with chronic heart failure. *Journal of the American College of Cardiology*.

[81] Arutyunov G. P., Kostyukevich O. I., Serov R. A., Rylova N. V., Bylova N. A. (2008). Collagen accumulation and dysfunctional mucosal barrier of the small intestine in patients with chronic heart failure. *International Journal of Cardiology*.

[82] Cui X., Ye L., Li J. (2018). Metagenomic and metabolomic analyses unveil dysbiosis of gut microbiota in chronic heart failure patients. *Scientific Reports*.

[83] Kamo T., Akazawa H., Suda W. (2017). Dysbiosis and compositional alterations with aging in the gut microbiota of patients with heart failure. *PLoS One*.

[84] Marques F. Z., Nelson E., Chu P.-Y. (2017). High-fiber diet and acetate supplementation change the gut microbiota and prevent the development of hypertension and heart failure in hypertensive mice. *Circulation*.

[85] Martin F. P. J., Wang Y., Sprenger N. (2008). Probiotic modulation of symbiotic gut microbial-host metabolic interactions in a humanized microbiome mouse model. *Molecular Systems Biology*.

[86] Shi K., Wang F., Jiang H. (2014). Gut bacterial translocation may aggravate microinflammation in hemodialysis patients. *Digestive Diseases and Sciences*.

[87] Tang W. H. W., Wang Z., Levison B. S. (2013). Intestinal microbial metabolism of phosphatidylcholine and cardiovascular risk. *New England Journal of Medicine*.

[88] Suzuki T., Heaney L. M., Bhandari S. S., Jones D. J. L., Ng L. L. (2016). TrimethylamineN-oxide and prognosis in acute heart failure. *Heart*.

[89] Schiattarella G. G., Sannino A., Toscano E. (2017). Gut microbe-generated metabolite trimethylamine-N-oxide as cardiovascular risk biomarker: a systematic review and dose-response meta-analysis. *European Heart Journal*.

[90] Organ C. L., Otsuka H., Bhushan S. (2016). Choline diet and its gut microbe-derived metabolite, trimethylamine N-oxide, exacerbate pressure overload-induced heart failure. *Circulation: Heart Failure*.

[91] Jia Q., Li H., Zhou H. (2019). Role and effective therapeutic target of gut microbiota in heart failure. *Cardiovascular Therapeutics*.

[92] Mayerhofer C. C. K., Ueland T., Broch K. (2017). Increased secondary/primary bile acid ratio in chronic heart failure. *Journal of Cardiac Failure*.

[93] Tang W. H. W., Li D. Y., Hazen S. L. (2019). Dietary metabolism, the gut microbiome, and heart failure. *Nature Reviews Cardiology*.

[94] Eblimit Z., Thevananther S., Karpen S. J. (2018). TGR5 activation induces cytoprotective changes in the heart and improves myocardial adaptability to physiologic, inotropic, and pressure-induced stress in mice. *Cardiovascular Therapeutics*.

[95] Fleischer J., Bumbalo R., Bautze V., Strotmann J., Breer H. (2015). Expression of odorant receptor Olfr78 in enteroendocrine cells of the colon. *Cell and Tissue Research*.

[96] Pluznick J. (2014). A novel SCFA receptor, the microbiota, and blood pressure regulation. *Gut Microbes*.

[97] De La Cuesta-Zuluaga J., Mueller N. T., Alvarez-Quintero R. (2018). Higher fecal short-chain fatty acid levels are associated with gut microbiome dysbiosis, obesity, hypertension and cardiometabolic disease risk factors. *Nutrients*.

[98] Aguilar E. C., Santos L. C. d., Leonel A. J. (2016). Oral butyrate reduces oxidative stress in atherosclerotic lesion sites by a mechanism involving NADPH oxidase down-regulation in endothelial cells. *The Journal of Nutritional Biochemistry*.

[99] Aguilar E. C., Leonel A. J., Teixeira L. G. (2014). Butyrate impairs atherogenesis by reducing plaque inflammation and vulnerability and decreasing NF-*κ*B activation. *Nutrition, Metabolism and Cardiovascular Diseases*.

